# Detection of novel biomarkers for early detection of Non-Muscle-Invasive Bladder Cancer using Competing Endogenous RNA network analysis

**DOI:** 10.1038/s41598-019-44944-3

**Published:** 2019-06-10

**Authors:** Morteza Kouhsar, Sadegh Azimzadeh Jamalkandi, Ali Moeini, Ali Masoudi-Nejad

**Affiliations:** 1 Laboratory of Systems Biology and Bioinformatics (LBB), Institute of Biochemistry and Biophysics (IBB), University of Tehran, Tehran, Iran; 2 Chemical Injury Research Center, Systems Biology and Poisonings Institute, Tehran, Iran; 3 Faculty of Engineering Sciences, College of Engineering, University of Tehran, Tehran, Iran

**Keywords:** Urological cancer, Biochemical networks

## Abstract

Bladder Cancer (BC) is one of the most common cancers in the world. Recent studies show that non-coding RNAs such as lncRNAs and circRNAs play critical roles in the progression of this cancer, but their regulatory relationships and functions are still largely unknown. As a new regulatory process within the cell, the coding and non-coding RNAs compete with each other to sponge their target miRNAs. This mechanism is described as “the competing endogenous RNA (ceRNA) hypothesis” which provides a new perspective to understand the regulation of gene expression in health and diseases such as cancer. In this study, to investigate the role of non-coding RNAs in BC, a new approach was used to reconstruct the ceRNA network for Non-Muscle Invasive Bladder Cancer (NMIBC) based on the expression data of coding and non-coding genes. Analysis of ceRNA networks in the early stage of BC led to the detection of an important module containing the lncRNA MEG3 as the central gene. The results show that the lncRNAs CARMN, FENDRR and ADAMTS9-AS2 may regulate MEG3 in NMIBC through sponging some important miRNAs such as miR-143-3p, miR-106a-5p and miR-34a-3p. Also, the lncRNA AC007608.2 is shown to be a potential BC related lncRNA for the first time based on ceRNA stage-specific network analysis. Furthermore, hub and altered genes in stage-specific and between stage networks led to the detection of hsa_circ_0017586 and hsa_circ_0001741 as novel potential circRNAs related to NMIBC. Finally, the hub genes in the networks were shown to be valuable candidates as biomarkers for the early stage diagnosis of BC.

## Introduction

Bladder Cancer (BC) is one of the most common cancers in men and women. This cancer is the 10th common cancer worldwide with about 200,000 people dying every year^1^. It has been estimated that there were 79,030 new cases of BC and 16,870 BC deaths in the United States in 2017^2^. In China, the mortality rate of BC patients has significantly increased in recent years, and 32,900 deaths were estimated in 2015^3^. Furthermore, due to the complex progression process and molecular interactions linked to the disease, the mechanism of BC remains poorly understood^4^.

Recent studies demonstrate that non-coding RNAs are closely related to BC progression and tumorigenesis^5,6^. Non-coding RNAs construct 80–90 percent of human transcripts^7^. This type of RNA is involved in many gene regulation processes in the cell, and any change in these processes can trigger complex disorders especially cancer^8^. Coding and non-coding RNAs regulate the expression of each other through common miRNAs that target them. This mechanism is known as the competing endogenous RNA (ceRNA)^9,10^. More precisely, transcripts with the same miRNA response elements (MREs) that are targeted by common miRNAs compete with each other to decoy or sponge shared miRNAs (these transcripts are called ceRNAs)^9^. This competition affects gene expression in the cell. Recent studies have shown that ceRNA interactions control many important biological processes and alteration in these interactions may cause diseases such as cancer^11–13^.

Based on previous studies, long non-coding RNAs (lncRNAs), circular RNAs (circRNAs), microRNAs (miRNAs) and transcribed pseudogenes are the most important non-coding RNAs involved in the ceRNA mechanism^10,14^. lncRNAs are RNAs longer than 200 nucleotides and play pivotal roles in many complex diseases^15^. In the past decade, the role of many lncRNAs has been studied in various cancer types. For example, lncRNA H19 is involved in metastasis, tumor initiation, and progression of many cancers^16^. HOTAIR, a well-known lncRNA, is overexpressed in lung cancer and correlates with its metastasis and poor prognosis^17^. Another type of non-coding RNA with very important regulatory functions are circRNAs^18^. These RNAs are constructed as a result of back-splicing from pre-mRNAs^19^. The conservation, stability, and tissue specificity properties of circRNAs make them valuable candidates for biomarker discovery in cancer research^18,20^. For instance, circRNA hsa_circ_0003221 promotes the proliferation and migration of BC cells^21^, and the expression of circRNA Hsa_circ_0000190 correlates with tumor size, metastasis, and disease stage of gastric cancer^20^. The pivotal non-coding RNAs in the ceRNA hypothesis are miRNAs. These non-coding RNAs are involved in post-transcriptional regulatory processes. They bind to the 3′ untranslated region (3′UTR) of mRNAs and prevent translation^22^. miRNAs dysregulation affects many critical biological processes such as cell proliferation, migration, differentiation, and apoptosis^23^. The expression of miRNAs significantly change in many cancerous tissues^24^, and these non-coding RNAs can act as both tumor suppressor or oncogene in cancer^25^.

The ceRNA interactions can be modeled as a network in which each node represents a ceRNA, and each interaction represents the competing relationship between two ceRNAs based on common miRNAs as mediators^14^. Reconstructing and analysis of such networks is a very fascinating approach to identify key genes in complex diseases to understand disease mechanism. Since the function of many non-coding RNAs (especially lncRNAs and circRNAs) in cancer remain unclear, the ceRNA network analysis can be helpful to discover novel cancer related non-coding RNAs as biomarkers or therapeutic targets. To understand the post-translation mechanism and non-coding RNA roles in BC, the ceRNA network analysis has been a desirable approach in recent years^4,6,7,26^. For instance, Zhu *et al*. proposed six lncRNAs as potential biomarkers for BC based on ceRNA network analysis^4^. Also, they found one miRNA and six differentially expressed mRNAs that correlated with BC patient survival rates. The ceRNA network reconstructed by Li *et al*. revealed that lncRNA MIR194-2HG, AATBC, and circRNA PGM5 could sponge the BC related miRNAs^6^. In^7^ constructing and analyzing the ceRNA network indicated that lncRNA H19 and circRNA MYLK compete with each other to decoy miRNA-29a-3p and increase the expression of its target genes DNMT3B, VEGFA and ITGB1. Moreover, in the same study, Wang *et al*. reconstruct the BC related ceRNA network based on 322 muscle-invasive bladder cancer tissues and 19 normal tissues^26^. Consequently, they proposed 5 BC related lncRNAs as potential biomarkers and therapeutic targets.

Although many studies have assessed the role of non-coding RNAs in BC, the molecular mechanism remain mostly unclear. To tackle this problem, in this study, we reconstruct and analyze a ceRNA network based on lncRNA, circRNA, transcribed pseudogene, and mRNA expression data from two early-stage urothelial carcinomas. A new computational approach was used to select disease-related mRNAs. Consequently, the stage-specific and the between stage ceRNA networks were reconstructed and examined. Finally, the hub nodes in the networks were analyzed as candidate prognostic biomarkers to detect BC at the early stages.

## Results

### Gene set enrichment

We selected the disease-related mRNAs based on their expression and interaction at the protein level for further analysis. Two approaches were used to this end: the PPR algorithm and the DNR approach (Eq. 2). Ten percent of the mRNAs with the highest rank (605 mRNAs) were selected in each approach, and about 70% of mRNAs chosen by the two methods were the same.

**Figure 1:**
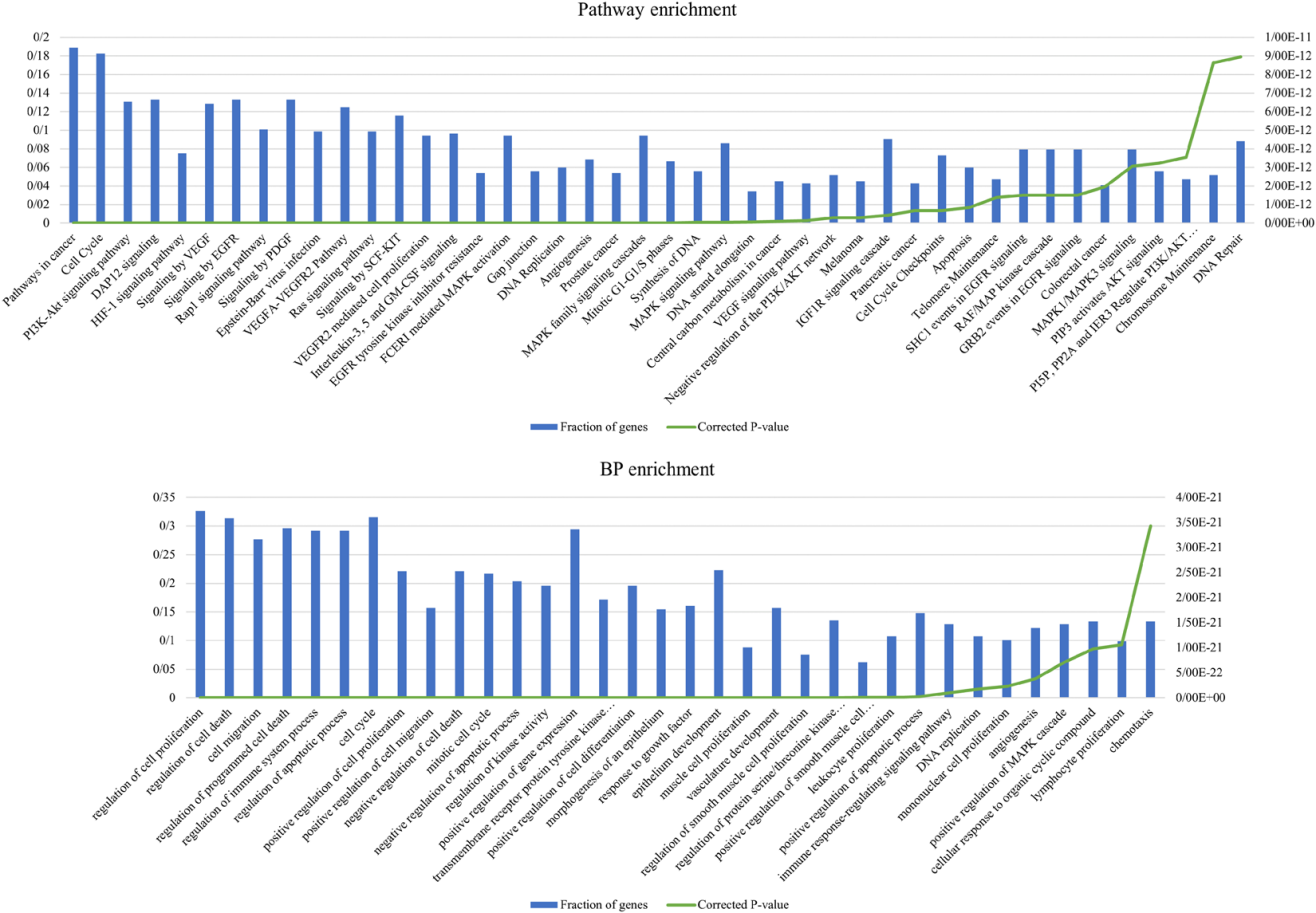
Some of the most critical enriched pathways and processes belonging to the mRNAs selected by DNR approach that they are not in the DEGs set.

To evaluate our new approach for selecting cancer-related mRNAs, we extracted those genes selected by our method that they were not in the DEGs set. Subsequently, the biological process, pathway, and disease enrichment analysis were applied to these extracted genes (Supplementary Files 2–4). The results show that the selected genes were associated to critical cancer-related pathways and processes such as “PI3K-Akt signaling pathway”, “Rap1 signaling pathway”, “Ras signaling pathway”, “regulation of cell proliferation”, “programmed cell death”, “cell cycle” and “DNA Repair” (Fig. 1, Supplementary File 1 and Fig. S1). Furthermore, we investigated the relationship between these genes and BC using PubMed enrichment analysis in ToppFun and literature search. For the genes selected by DNR and PPR method, 62 and 45 articles about BC were found, respectively. Also, 107 and 81 genes of the genes selected by DNR and PPR respectively were studied in those mentioned articles (Supplementary File 5). These results show that there are many BC related genes in our samples that are not differentially expressed, but our new approach may be able to find them. For example, Michiels *et al*.^27^ investigated the role of 85 DNA repair genes in BC. Among the genes significantly related to BC in this study, six genes were found by our approach (CDKN2A, FANCD2, LIG1, POLR2K, RFC2, and RFC5). In a recent study, Books *et al*.^28^ experimentally found a significant association between the expression of COL1A1 and COL1A2 mRNAs and NMIBC. These genes are also found by the PPR method. MDM2, HMOX1, and SDC1 are other examples of the NMIBC related genes^29,30^ that are not differentially expressed in the samples but are found by our ranking methods.

### Stage-specific ceRNA networks

Considering selected key mRNAs based on PPR and DNR methods, two ceRNA networks for each stage of BC were reconstructed (Supplementary File 1, Figs S2 and S3):

- DNR-Ta: the network of ceRNAs that was reconstructed based on the mRNAs selected by the DNR method based on stage Ta expression data.
- DNR-T1: the network of ceRNAs that was reconstructed based on the mRNAs selected by the DNR method based on stage T1 expression data.
- PPR-Ta: the network of ceRNAs that was reconstructed based on the mRNAs selected by the PPR method based on stage Ta expression data.
- PPR-T1: the network of ceRNAs that was reconstructed based on the mRNAs selected by the PPR method based on stage T1 expression data.

Table 1 shows the global properties of these networks. The degree distribution of the networks follows the power low mode (Supplementary File 1, Figs S2 and S3). It is clear that the networks are scale-free and their connections are not organized randomly.

**Table 1:** Global properties of the stage-specific networks.

Network	# nodes	# edges	# mRNAs	# lncRNAs or Pseudogenes	# circRNAs
DNR-Ta	1692	32478	529	910	253
PPR-Ta	1655	31311	494	909	252
DNR-T1	1295	13899	491	624	180
PPR-T1	1283	15010	469	632	182

#### Stage-specific modules

**Figure 2:**
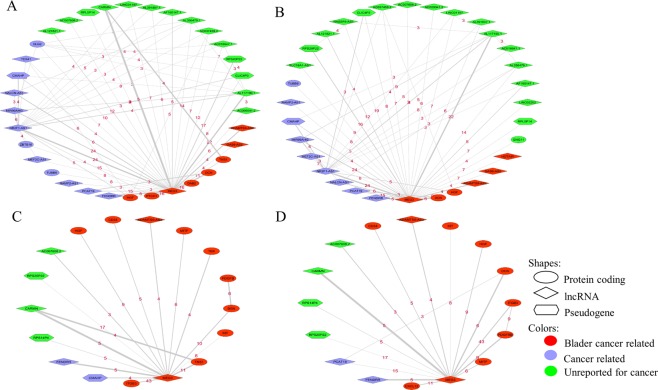
MEG3 module in stage-specific ceRNA networks ((**A**): DNR-Ta, (**B**): PPR-Ta, (**C**): DNR-T1, (**D**): PPR-T1). The thickness of the edges shows the correlation, and the number on each edge is the number of shared miRNAs between two genes.

To find important functional blocks in each stage-specific network, the MCL algorithm was used to cluster the networks and detect modules. Consequently, the ToppFun web tool was used to perform GO, pathway, and disease enrichment analysis on the modules. Considering the modules with a size larger than three nodes, 55 and 83 modules were detected in DNR-T1 and DNR-Ta networks, respectively (48 and 90 modules in PPR-T1 and PPR-Ta networks, respectively). Although the enrichment tools could not find any significant process and pathway for many non-coding modules, we found some significant cancer-related pathways and processes in the modules based on enrichment analysis on coding genes (Supplementary Files 6–9). The most important modules (called MEG3 modules) detected in stage-specific networks are the modules depicted in Fig. 2.

As is shown in Fig. 2, many of the genes in the modules are the same (almost 75–80 percent of genes in modules detected by two approaches in each stage are common). This shows that in fact, we found one module by two approaches, altered when the disease has progressed from stage Ta to T1. Searching in the literature shows that there are many cancer-related genes in this module (Supplementary File 1, Table S1). Also, the biological process and pathway enrichment analysis of the protein-coding genes in this module showed that it is involved in many important processes and pathways in human cells (Supplementary File 10). The genes in this module could be interesting candidates for therapeutic targets.

#### Stage-specific key genes

Key genes in the stage-specific networks were detected using the degree, and betweenness centrality measures. The degree of each gene in the network demonstrates the number of genes that are connected with it and the betweenness centrality measures the amount of shortest path that passes from the genes. Therefore, the genes with high degree and betweenness centralities can be important because these genes compete with many other genes and play a pivotal role to construct pathways in the network, respectively. Accordingly, we selected the top 10 genes with the highest degree and betweenness centrality in each stage-specific network as key genes and analyzed cancer relativity of them based on literature searches (Tables 2 and 3).

**Table 2:** Key nodes in stage Ta networks based on Degree and Betweenness centrality measures.

Gene name	Gene type	DNR-Ta network*	PPR-Ta network*	Some related cancers	Reference (PMID)
NRAS	Protein coding	D, B	D, B	**Bladder**, Colorectal, Melanoma	21072204 22820081 28074351
PDIA6	Protein coding	D, B	—	**Bladder**, Breast	27760590 26125904
CCNG1	Protein coding	D, B	—	**Bladder**, Ovarian	27982046 25981880
hsa_circ_0000591	circRNA	D	D	—	—
hsa_circ_0000592	circRNA	D	D, B	—	—
RPLP0P6	Pseudogene	D, B	D, B	—	—
PARPBP	Protein coding	D	D	Pancreatic	23436799
CCNT2	Protein coding	D	—	leukemia	28409330
MSH6	Protein coding	D	D, B	**Bladder**, colorectal	20591884 21642682
hsa_circ_0003221	circRNA	D	D	**Bladder**	29125888
KCNQ1OT1	lncRNA	B	B	Breast, Lung	30157476 28600629
PWAR6	lncRNA	B	B	Glioma, Breast	30472640 30297886
WEE1	Protein coding	B	B	Breast, Leukemia, Melanoma, Brain	27427153
HCG18	lncRNA	B	B	**Bladder**	30426533
AC015813.1	lncRNA	B	B	—	—
NCAPD2	Protein coding	B	—	**Bladder**	21982874
RFC3	Protein coding	—	D	Breast, Ovarian, Esophageal	27888707 26464638 22328562
CCNA2	Protein coding	—	D	**Bladder**, Breast, Colorectal	30138038 24622579 30464611
hsa_circ_0017586	circRNA	—	D	—	—
NAMPTP1	Pseudogene	—	B	—	—

*D: Degree, B: Betweenness.

**Table 3:** Key nodes in stage T1 networks based on Degree and Betweenness centrality measures.

Gene name	Gene type	DNR-T1 network*	PPR-T1 network*	Some related cancers	Reference (PMID)
CCNA2	Protein coding	D	D	**Bladder**, Breast, Colorectal	30138038 24622579 30464611
CCNB1	Protein coding	D	D	**Bladder**, Breast, Gastric	30170380 28821558 30518842
PARPBP	Protein coding	D	D	Pancreatic	23436799
NCAPD2	Protein coding	D	D	Ovarian	21423607
RFC3	Protein coding	D	D	Breast, Ovarian, Esophageal	27888707 26464638 22328562
MCM7	Protein coding	D	D	**Bladder**, Lung	23201130 27797825
KPNA2	Protein coding	D	D	**Bladder**, Lung, Colorectal	27611951 27009856 26663089
ASF1B	Protein coding	D	D	Breast	21179005
LMNB1	Protein coding	D	—	Liver, Cervical	19522540 30394198
CDC25A	Protein coding	—	D	**Bladder**, Breast, Ovarian	29246203 30443946 30405749
NUP214	Protein coding	B	—	Leukemia	25120641
RPLP0P6	Pseudogene	B	B	—	—
NR2F1-AS1	lncRNA	B	—	Hepatocellular carcinoma	29602203
YWHAH	Protein coding	B	—	Glioma	26370624
GAPDHP1	Pseudogene	D, B	B	—	—
NRAS	Protein coding	B	B	**Bladder**, Colorectal, Melanoma	21072204 22820081 28074351
LINC01355	lncRNA	B	—	Lung	28949095
GLI2	Protein coding	B	—	**Bladder**, Gastric	27465044 28975979
CCNT2	Protein coding	B	—	leukemia	28409330
EEF1A1P13	Pseudogene	B	—	—	—
FASN	Protein coding	—	D	**Bladder**, Breast	30221356 28922023
ANXA2P2	Pseudogene	—	B	—	—
HCG18	lncRNA	—	B	**Bladder**	30426533
NCOR2	Protein coding	—	B	Lung	29764865
COL1A2	Protein coding	—	B	**Bladder**, Gastric	27655672 28401451
MEG3	lncRNA	—	B	**Bladder**, Gastric, Esophageal, Glioma, Breast, Ovarian	30461333 30417553 30310931 28975980 28539329 30389444
ENO1	Protein coding	—	B	Pancreatic, Gastric, Colorectal	28086938 29986635 26097998
CDC25B	Protein coding	—	B	**Bladder**, Endometrial, Gastric	29234286 29353204 27863420

*D: Degree, B: Betweenness.

**Figure 3:**
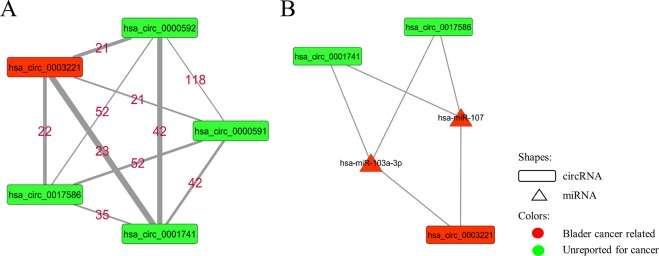
The module constructed from 5 hub circRNAs in Ta networks.

As shown in Table 3, no circRNA is detected as a hub in T1 networks, but in Ta networks, the circRNAs are seen in the hub nodes set. If 13 and 12 nodes with the highest degree are considered in PPR-Ta and DNR-Ta networks respectively, five circRNAs (hsa_circ_0000591, hsa_circ_0000592, hsa_circ_0003221, hsa_circ_0017586, hsa_circ_0001741) are seen as hub nodes in both networks. hsa_circ_0000591, hsa_circ_0000592, and hsa_circ_0003221 are derived from the PTK2 gene and hsa_circ_0017586, and hsa_circ_0001741 are derived from GDI2 and TNPO3, respectively. Interestingly, all of these transcripts interact with each other in Ta networks (Fig. 3A). Furthermore, the circRNA hsa_circ_0003221 has been recently reported as a novel biomarker in BC that promotes the migration and proliferation of BC cells^21^. hsa_circ_0003221, hsa_circ_0017586 and hsa_circ_0001741 share 2 miRNAs that are involved in multiple cancers including BC (Fig. 3B)^31–38^.

### Between stage networks

**Figure 4:**
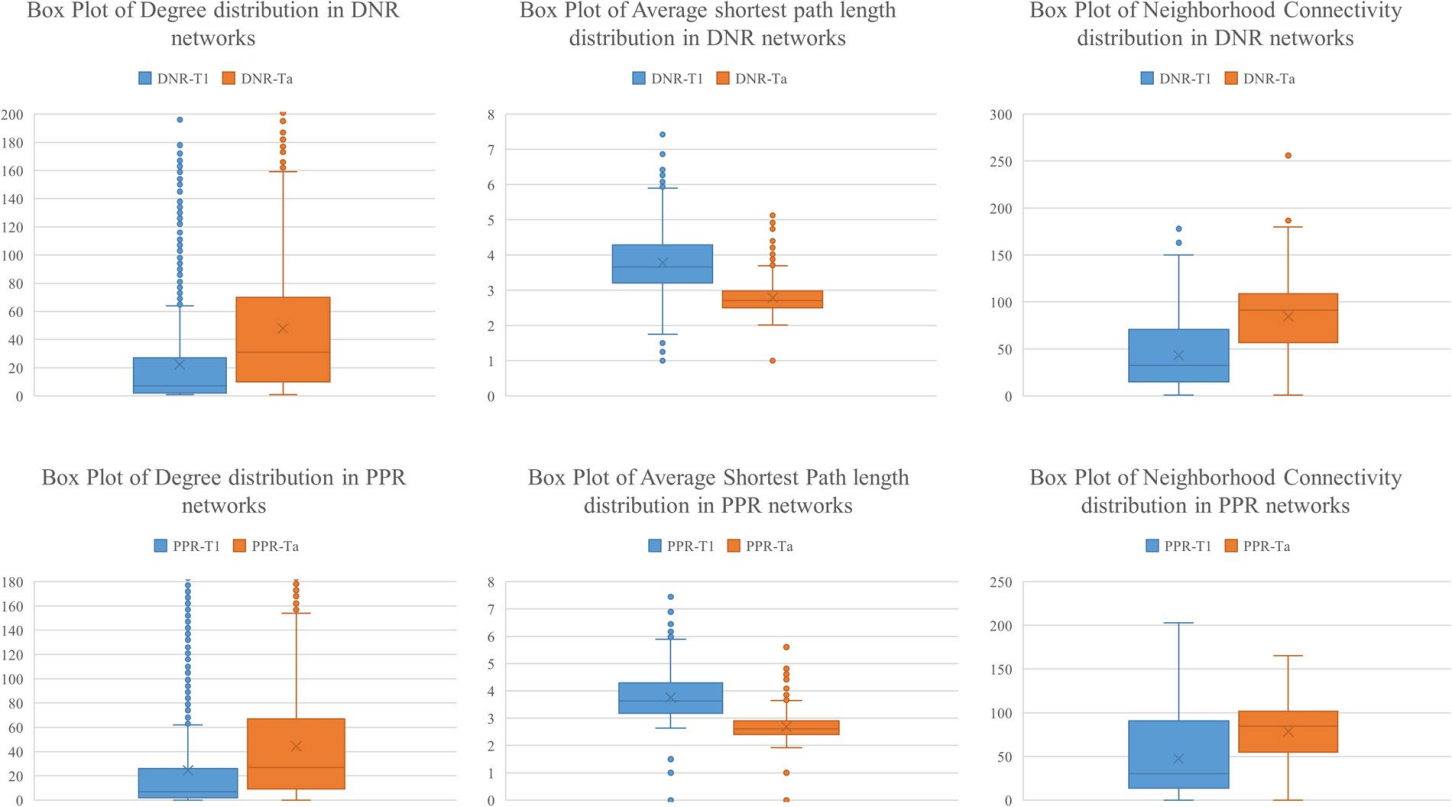
Comparing the connection between genes in Ta and T1 networks. The node degree, average shortest path and neighborhood connectivity distributions in all stage-specific networks show that many of connections have been lost in T1 networks relative to Ta.

To investigate how the connections of the ceRNAs change between stage Ta and T1, we compared the degree, shortest path and neighborhood connectivity of the genes in stage-specific networks (Fig. 4). As shown in Fig. 4, the degree and neighborhood connectivity of the genes in Ta networks are higher than T1. Also, the average shortest path of the genes in T1 networks is higher than Ta. These results show that many of the ceRNAs connections are lost when the tumor progresses from Ta to T1 stage. To determine which connection is altered and which genes have the most changes in their connections, we reconstructed the between stage networks through calculating the difference of edges. Two network types were defined: Lose Interaction Network (*LIN* = *Ta* − *T*1) and Gain Interaction Network (*GIN* = *Ta* − *T*1).

Subsequently, the most important genes in these networks (genes with the most connection changes from stage Ta to T1) were detected using degree and betweenness centrality measures (top 10 genes with the highest rank based on each measure were selected). The results show that most of the important genes in the between stage networks, especially the genes with the most altered connections (high degree or hub genes) are critical in cancer (Tables 4 and 5). These genes could be potential biomarkers for bladder cancer diagnosis. For example, circRNA hsa_circ_0003221 is one of the hub genes in DNR-LIN and PPR-LIN networks. Recently, Xu *et al*.^21^ have demonstrated that this circRNA promotes the proliferation and migration of BC cells.

**Table 4:** Key nodes in stage LIN networks based on Degree and Betweenness centrality measures.

Gene name	Gene type	DNR-LIN network*	PPR-LIN network*	Some related cancers	Reference (PMID)
hsa_circ_0000591	circRNA	D, B	D, B	—	—
CCNT2	Protein coding	D, B	—	Leukemia	28409330
hsa_circ_0000592	circRNA	D	D, B	—	—
NRAS	Protein coding	D, B	D, B	**Bladder**, Colorectal, Melanoma	21072204 22820081 28074351
WEE1	Protein coding	D, B	D, B	Breast, Leukemia, Melanoma, Brain	27427153
CCNG1	Protein coding	D, B	—	**Bladder**, Ovarian	27982046 25981880
hsa_circ_0001741	circRNA	D	D	—	—
hsa_circ_0017586	circRNA	D	D	—	—
hsa_circ_0007646	circRNA	D	D	Hypopharyngeal squamous cell carcinoma, Pancreatic	28514762 29922161
hsa_circ_0003221	circRNA	D	D	**Bladder**	29125888
MDM2	Protein coding	B	B	**Bladder**, Ovarian	28498468 28817834
PWAR6	lncRNA	B	B	Glioma, Breast	30472640 30297886
KCNQ1OT1	lncRNA	B	B	Breast, Lung	30157476 28600629
hsa_circ_0008345	circRNA	—	D	—	—
AC015813.1	lncRNA	B	B	—	—
IGF1R	Protein coding	B	—	**Bladder**, Colon	27470393 28882129
NEAT1	lncRNA	—	B	**Bladder**, thyroid	30349370 30596336
GABPB1-AS1	lncRNA	—	B	—	—
hsa_circ_0011536	circRNA	—	D	—	—

*D: Degree, B: Betweenness.

**Table 5:** Key nodes in stage GIN networks based on Degree and Betweenness centrality measures.

Gene name	Gene type	DNR-GIN network*	PPR-GIN network*	Some related cancers	Reference (PMID)
FADD	Protein coding	D, B	—	Pancreatic, Lung	28454341 30190126
PSMC4		D	D	Breast	18042273
GAPDHP1	Pseudogene	D, B	D	—	—
PDIA5	Protein coding	D	—	Melanoma	25912252
SLC25A1	Protein coding	D	—	Lung	29651165
TFRC	Protein coding	D	D	Melanoma	28551638
SNRPA	Protein coding	D	—	Gastric	30039889
MYC	Protein coding	D, B	D, B	**Bladder**, Prostate	29463565 27159573
MCM3	Protein coding	D	D	Hepatocellular Carcinoma	30363964
HSPA1A	Protein coding	D		**Bladder**, Ovarian	23874968 26868087
NR3C1	Protein coding	B	—	**Bladder**, Gastric	29991691 29285253
NUP214	Protein coding	B	—	Leukemia	25120641
NR2F1-AS1	lncRNA	B	B	Hepatocellular carcinoma	29602203
GLI2	Protein coding	B	—	**Bladder**, Gastric	27465044 28975979
NCOR2	Protein coding	B	—	Lung	29764865
ADCY7	Protein coding	B	B	Leukemia	26220344
FASN	Protein coding	—	D, B	**Bladder**, Breast	30221356 28922023
CBX2	Protein coding	—	D	Ovarian	30478317
CDC25B	Protein coding	—	D, B	**Bladder**, Endometrial, Gastric	29234286 29353204 27863420
ADCY3	Protein coding	—	D	Gastric	24113161
DAG1	Protein coding	—	D	Leukemia	28591567
STAT1	Protein coding	—	B	**Bladder**, Breast, Lung, Melanoma, Gastric, Colorectal, Esophageal	27080594 28950072
ANXA2P2	Pseudogene	—	B	—	—
CDK6	Protein coding	—	B	**Bladder**, Esophageal	30362519 30551480
IL10	Protein coding	—	B	**Bladder**, Melanoma, Lung	27987237 26188281
PART1	lncRNA	B	—	Esophageal, Lung, Prostate	30049286 28819376 29261512
LINC02607	lncRNA	—	B	—	—

*D: Degree, B: Betweenness.

### Potential biomarker detection

As mentioned in section 2.8, we analyzed the hub nodes (nodes with the highest degree) as candidate biomarkers to investigate which hubs were able to separate Ta and T1 samples based on their expression patterns. Since the expression of circRNAs relative to other RNAs is very low in the samples (Supplementary File 1, Fig. S4), we analyzed circRNAs separately. Overall, 48 gene sets from 8 networks were analyzed (16 circRNA sets and 32 other RNA sets from DNR and PPR networks). Note that there were no circRNAs as a hub in the GIN and T1 networks.

None of the circRNA sets were able to separate Ta and T1 samples significantly. Among all circRNA hubs, five circRNAs (Fig. 3A) with the highest degree in Ta networks (DNR-Ta and PPR-Ta) showed approximately good clustering, but validation results demonstrated that these genes are not able to separate samples based on their expression pattern or maybe our clustering method is not sensitive enough for the circRNAs (Supplementary File 1, Fig. S5). These transcripts are seen in the top ten circRNAs with the highest degree in all networks (except T1 and GIN networks that do not have any circRNAs in their highest degree genes).

**Figure 5:**
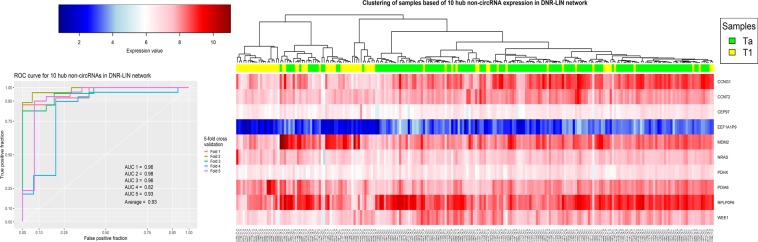
The clustering and Validation results for ten non-circRNAs with the highest degree in the DNR-LIN network.

**Figure 6:**
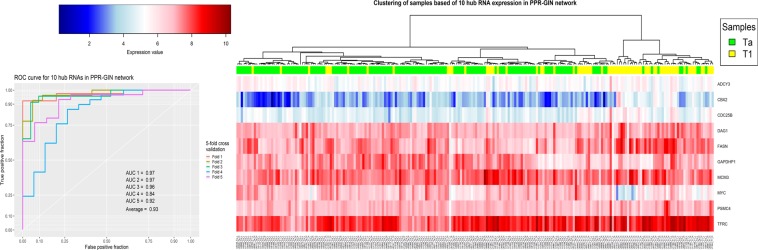
The clustering and Validation results for ten genes with the highest degree in the PPR-GIN network.

In the LIN networks, the set of 10 non-circRNAs with the highest degree in the DNR-LIN network showed an acceptable clustering result on all samples (Fig. 5). The validation results for these genes were also satisfactory and the average AUC in the 5-fold cross validation was 0.93 for these genes. For the GIN networks, the best clustering result was obtained from the 10 RNAs with the highest degree in the PPR-GIN network (Fig. 6).

**Figure 7:**
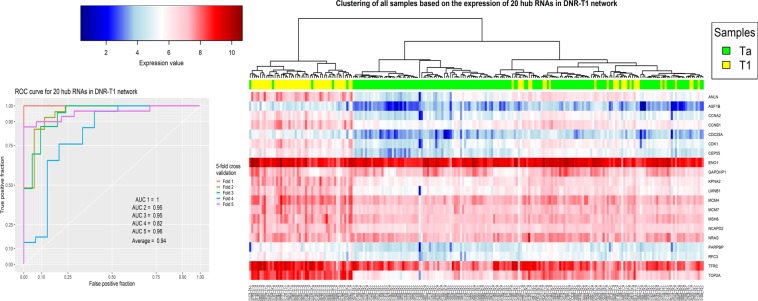
The clustering and Validation results for 20 genes with the highest degree in the DNR-T1 network.

The clustering result for 20 genes with the highest degree in DNR-T1 networks was interesting, and the validation showed that these genes could be a bona fide potential biomarker set to separate Ta and T1 bladder cancer stages from each other (Fig. 7).

**Figure 8:**
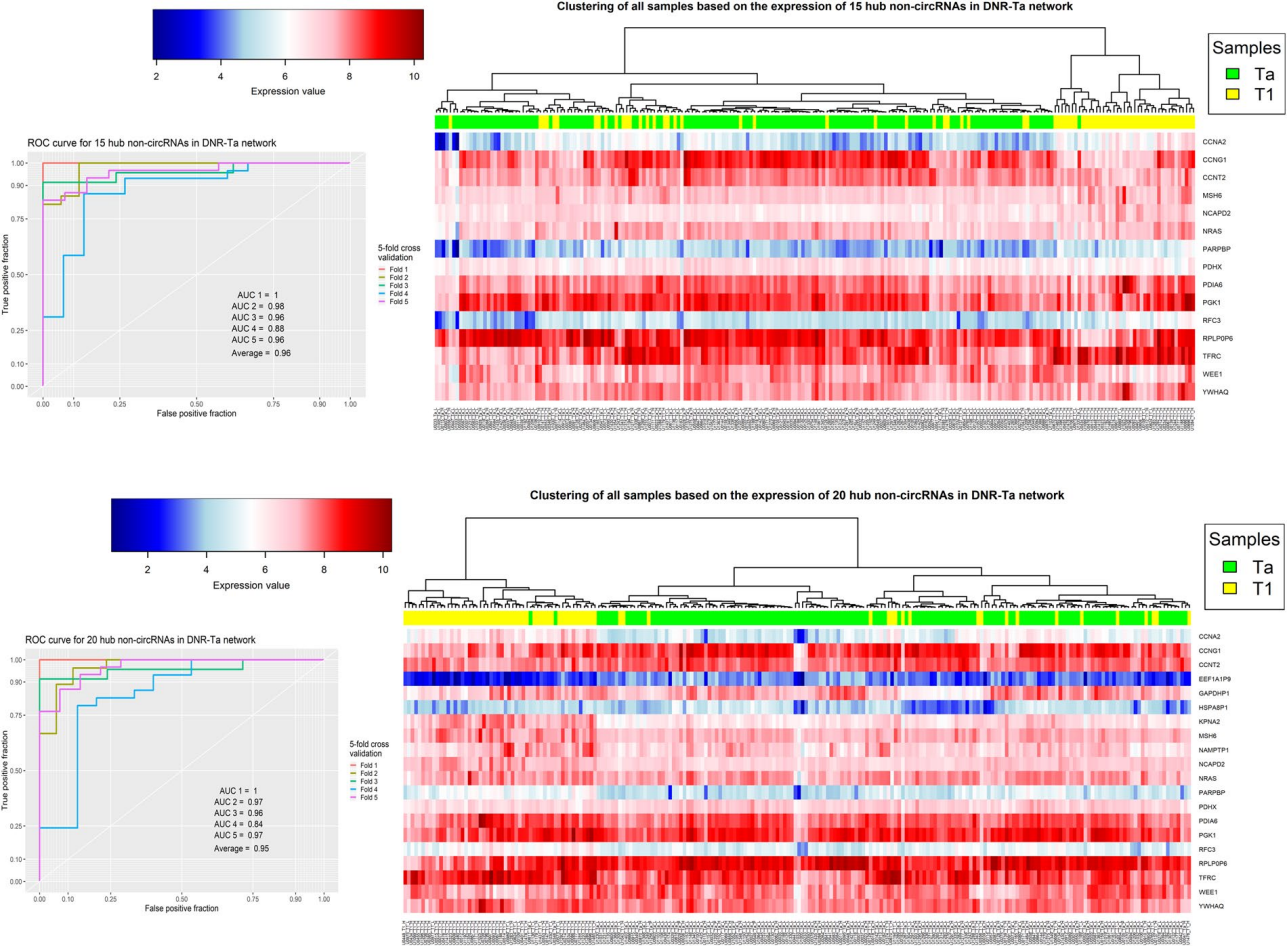
The clustering and validation results for 15 and 20 genes with the highest degree in DNR-Ta network.

The results show that the hub genes in the Ta networks are interesting to further investigate as biomarkers. The best validation result was acquired from the set of 15 and 20 high degree non-circRNA genes in the DNR-Ta network (Fig. 8). The average AUCs for these sets in the 5-fold cross validation were 0.96 and 0.95, respectively. Approximately the same results were acquired from the PPR-Ta network (Supplementary File 1, Fig. S6).

## Discussion

Reconstructing the ceRNA networks based on differentially expressed genes is widely used in the researches but the expression difference is not the only key factor in the ceRNAs activity. For instance, a gene may decoy a large number of miRNAs through a large number of MREs while its expression may not be changed significantly^14^. To challenge those mentioned limitations and reconstruct ceRNA network for Non-Muscle Invasive Bladder Cancer (NMIBC), we used the expression data of four RNA types (mRNA, lncRNA, pseudogene, and circRNA) in stages of Ta and T1 in bladder cancer transcriptomics data. Additionally, we integrated gene expression data with PPI scaffold to select the ceRNAs based on both expression changes and interactions at the protein level.

The stage-specific network analysis led to the prediction of an important module, entitled the MEG3 module, containing some key cancer-related coding and non-coding genes. As shown in Fig. 2, indeed four modules in four networks were predicted but many of the genes in these modules are the same. More precisely, there is one module (detected by two computational approaches) that has its genes and interactions rewired when the disease progresses from stage Ta to T1. Many of the genes in the MEG3 module are cancer-related (more than 80% in T1 and more than 50% in Ta networks), and interestingly in T1 networks, all protein-coding genes detected in the module are bladder cancer-related. This proves that our suggested approach enjoys significant power to predict disease-related genes.

lncRNA MEG3 is the core gene in this module that interacts with all other genes (except PDGFB and AC018647.1 in DNR-T1 and PPR-Ta networks, respectively). This is a very important cancer-related lncRNA that acts as a tumor suppressor in many cancers including bladder cancer^5,39–41^. Strong correlated competing interactions are observed between this gene and some other bladder cancer-related genes in this module that has not yet been reported for NMIBS. For example, ADAMTS9-AS2 is a bladder cancer-related lncRNA^4^ that interacts with MEG3 in both Ta and T1 networks through sharing four miRNAs (hsa-miR-148b-3p, hsa-miR-143-3p, hsa-miR-574-5p and hsa-miR-4701-3p). Among these miRNAs, miR-574-5p is an important cancer-related miRNA that shows oncogenic function in thyroid, breast, and lung cancers^42,43^ but has been not reported in NMIBC. CARMN (or miR143HG) is another bladder cancer related lncRNA^44^ that has a strong connection with MEG3 (correlation 0.76) via sharing 5 miRNAs (hsa-miR-148b-3p, hsa-miR-106a-5p, hsa-miR-9-5p, hsa-miR-148a-3p, hsa-miR-501-3p). miR-106a-5p is significantly associated with tumor grade in NMIBC^45^ and miR-148a-3p suppresses Epithelial Mesenchymal Transition (EMT) and cell proliferation in bladder cancer^46^.

An example of a bladder cancer-related mRNA that is found in the MEG3 module is Decorin^47^. In our results, the lncRNA MEG3 competes with Decorin through sponging 6 miRNA including hsa-miR-122-5p, hsa-miR-484, hsa-miR-338-3p, hsa-miR-30a-3p, hsa-miR-30e-3p, hsa-miR-224-5p. miR-484 is a tumor suppressor miRNA between MEG3 and Decorin in our module. Also, the lncRNA ZFAS1 promotes colorectal cancer cell proliferation via sponging this miRNA^48^. Furthermore, ZFAS1 recently has been detected as an oncogenic lncRNA in BC^49^. Therefore, miR-484 can be introduced as an important NMIBC related miRNA.

FENDRR is another key lncRNA in MEG3 module. This gene has a suppressive function in non-small cell lung cancer, and downregulation of it correlates with poor prognosis in renal cell carcinoma^50,51^. We found just one study by Suqing *et al*.^52^ reporting it as a significantly downregulated lncRNA in bladder cancer. Five miRNAs are shared between FENDRR and MEG3 in our networks (hsa-miR-148a-5p, hsa-miR-106b-5p, hsa-miR-4705, hsa-miR-20a-5p, hsa-miR-34a-3p). Interestingly, all of these miRNAs (except miR-4705) are bladder cancer related^53–55^, and one of them (miR-34a-3p) has been reported as a biomarker of NMIBC recurrence^56^. These results indicate that the lncRNA FENDRR can potentially be a novel candidate lncRNA related to NMIBC.

As shown in Fig. 2, there are some cancer-related genes in the MEG3 module that highly correlate and interact with bladder cancer genes, but no report was found about the activity of these genes in bladder cancer. These genes are potentially related to NMIBC and should be investigated as future work. For instance, NR2F1-AS1 was strongly connected with MEG3 in Ta networks. This gene is relevant to oxaliplatin resistance in hepatocellular carcinoma^57^. Furthermore, some other genes in the module such as lncRNA AC007608.2 and AL117190.1 have a strong connection with MEG3 and some other cancer-related genes, but we did not find any report about their roles in cancer. These genes could be candidate genes to investigate their function in bladder cancer especially NMIBC. Taking a closer look at these interactions and shared miRNAs can help to select a better candidate gene. For instance, there are three shared miRNAs between AC007608.2 and MEG3 and one of them (miR-324-3p) is bladder cancer related^58^ and two others (miR- 365a-3p, miR-365b-3p) are involved in other cancer types^59,60^. Therefore, the lncRNA AC007608.2 is another novel candidate gene potentially related to NMIBC.

Change in ceRNA interactions is one of the key features of cancer^11,13^. Therefore, we reconstructed between-stage networks (LIN and GIN) by calculating the difference between the edge set of Ta and T1 networks. Subsequently, the top 10 hub genes (10 genes with the highest degree) in these networks were detected as the genes with the most connection rewiring when the disease progressed from Ta to T1 stage. In the LIN and Ta networks, five circRNAs had the highest degrees simultaneously (Fig. 3A). Among these hub circRNAs, hsa_circ_0003221, hsa_circ_0000591, and hsa_circ_0000592 were derived from one gene (PTK2), and hsa_circ_0003221 recently has been reported as a potential biomarker for bladder cancer that promotes proliferation and migration of cancer cells^21^. We did not find any report about the four other circRNAs in this set but closely looking in the module constructed by these transcripts revealed that there are only two important miRNAs (miR-103a-3p, miR-107) shared between hsa_circ_0003221, hsa_circ_0017586, and hsa_circ_0001741 (Fig. 3B). These two miRNAs have recently been reported as regulators to promote proliferation and migration of BC cells through ceRNA activity of circTCF25 and CDK6^31^. miR-107 is one of the most important miRNAs in cancer^36^. This miRNA is sponged by the lncRNA RP11-79H23.3 and regulates PTEN in BC cells^34^. This process can suppress BC development via inactivation of the PI3K/Akt signaling pathway^35^. The tumor suppressor function of miR-107 via ceRNA activity has also been demonstrated by Su *et al*. in another recent study^32^. Therefore, hsa_circ_0017586 and hsa_circ_0001741 may have oncogenic functions in BC through sponging miR-107. Furthermore, miR-103a-3p, another miRNA shared between the three mentioned circRNAs, is also involved in BC and some other cancer types such as Gastric and Glioma^33,37,38^.

The last analysis in this study was to evaluate the hub genes as potential biomarkers to design a diagnostic panel. To this end, the set of hub genes in each network were analyzed using hierarchical clustering with correlation based distance and SVM classifier with 5-fold cross validation (see section Materials and method). As shown in Figs 5–8 and Supplementary File 1, Fig. S6, the results show the power of hub genes to separate Ta and T1 samples based on their expression patterns.

The results of our study demonstrate that the selection of genes via a computational manner such as mapping the expression difference of genes on PPI data and ranking them based on their connection and expression could be an alternative approach to find significant disease-related genes. Also, the ceRNA hypothesis and its novel perspective to the gene regulatory system could be very helpful to understand complex disease mechanisms such as cancer.

## Material and Method

### Data collection

Total RNA sequencing of 457 Non-Muscle-Invasive Bladder Cancer (NMIBC) samples (348 samples in stage Ta and 109 samples in stage T1) were selected from the ArrayExpress database (http://www.ebi.ac.uk/arrayexpress)^61^ under accession number E-MTAB-4321^62^. circRNA expression data was obtained from Okholm *et al*.^63^.

### Preprocessing and normalization

Based on the ceRNA hypothesis, lncRNAs, transcribed pseudogenes, circRNAs, and mRNAs are four key RNA categories that act as ceRNAs^14,64^. Therefore, we selected only these types of RNAs for further analysis. The BioMart tool^65^ from the Ensembl genome browser (www.ensembl.org)^66^ was used to select the gene types.

In the next step, the genes with low expression in all samples were removed. To this end, we filtered out each gene that had an average count in all samples of less than one^67^. Furthermore, the genes with low standard deviation (lower than the first quartile of the standard deviation of all genes) in all samples were also filtered out. Additionally, the genes with a high standard deviation of their count in T1 or Ta stages were also filtered out (threshold was the third quartile of the standard deviation of all genes in each group).

Considering all samples of a stage of the disease, if the average Pearson correlation of a sample with other samples was less than the first quartile of Pearson correlations between all sample pairs, then this sample was ignored as a within stage outlier. After all preprocessing steps, 220 samples (148 Ta and 72 T1) were selected for further analysis. The ceRNA count data was normalized using the TMM method^68^ implemented in the edgeR package^69^ and the log-transformed normalized count table used for further analysis.

### Key mRNA selection

Generally, due to a large number of genes, the ceRNA networks are reconstructed based on Differentially Expressed Genes (DEGs), but it is obvious that DEGs are not the only actors in this process^14^. Therefore, we tried to find key disease-related mRNAs based on expression data and physical interactions at the protein level. To this end, firstly we applied differential expression analysis to get the fold change and p-value of all mRNAs (the edgeR package was used). After that, a combined weight was assigned to each mRNA based on Eq. 1:

1 $$w(g)=|{\rm{l}}{\rm{o}}{\rm{g}}({\rm{F}}{\rm{o}}{\rm{l}}{\rm{d}}\,{\rm{C}}{\rm{h}}{\rm{a}}{\rm{n}}{\rm{g}}{\rm{e}}({\rm{g}}))|\ast (\,-\,{\rm{l}}{\rm{o}}{\rm{g}}({\rm{c}}{\rm{o}}{\rm{r}}{\rm{r}}{\rm{e}}{\rm{c}}{\rm{t}}{\rm{e}}{\rm{d}}\,{\rm{P}}{\rm{\_}}{\rm{V}}{\rm{a}}{\rm{l}}{\rm{u}}{\rm{e}}({\rm{g}}))$$

where *g* is a protein-coding gene or mRNA in the data.

In the next step, the Protein-Protein Interactions (PPI) of all weighted mRNAs were extracted from the STRING database^70^ with a minimum interaction confidence score of 0.5. The weighted nodes in the PPI network were ranked based on their weights and connections. Finally, 10 percent of the nodes with the highest ranks were selected for further analyses. Two ranking methods were applied: the Personalized PageRank (PPR) algorithm^71^ (implemented by the igrapg package^72^) and our proposed method based on Eq. 2 (entitled Depth Neighborhood Ranking or DNR):

2 $$rank(g)=w(g)+\sum _{i=1}^{d}\frac{\sqrt{|{N}_{g}^{d}|}}{d}(\sum _{|{N}_{g}^{d}|}w({N}_{g}^{d}))$$

where *g* is a vertex in the PPI network (a protein-coding gene), *w*(*g*) is the weight of vertex *g* calculated by Eq. 1, ${N}_{g}^{d}$ is the set of neighbors of vertex *g* that the shortest path of g to them is equal to *d*.

In this new approach, we tried to add a fraction of total weights of the neighbors to the initial weight of each vertex. This fraction is the square root of the number of neighbors because the slope of the square root function grows softly by increasing the number of neighbors. Concerning “*d*” as a depth threshold, the effect of farther neighbors can be applied. However, the greater the distance from *g* the lower the effect as neighbors weights are divided by *d* threshold (The *d* threshold was set to 2 as default).

### ceRNA network reconstruction and Module Detection

**Figure 9:**
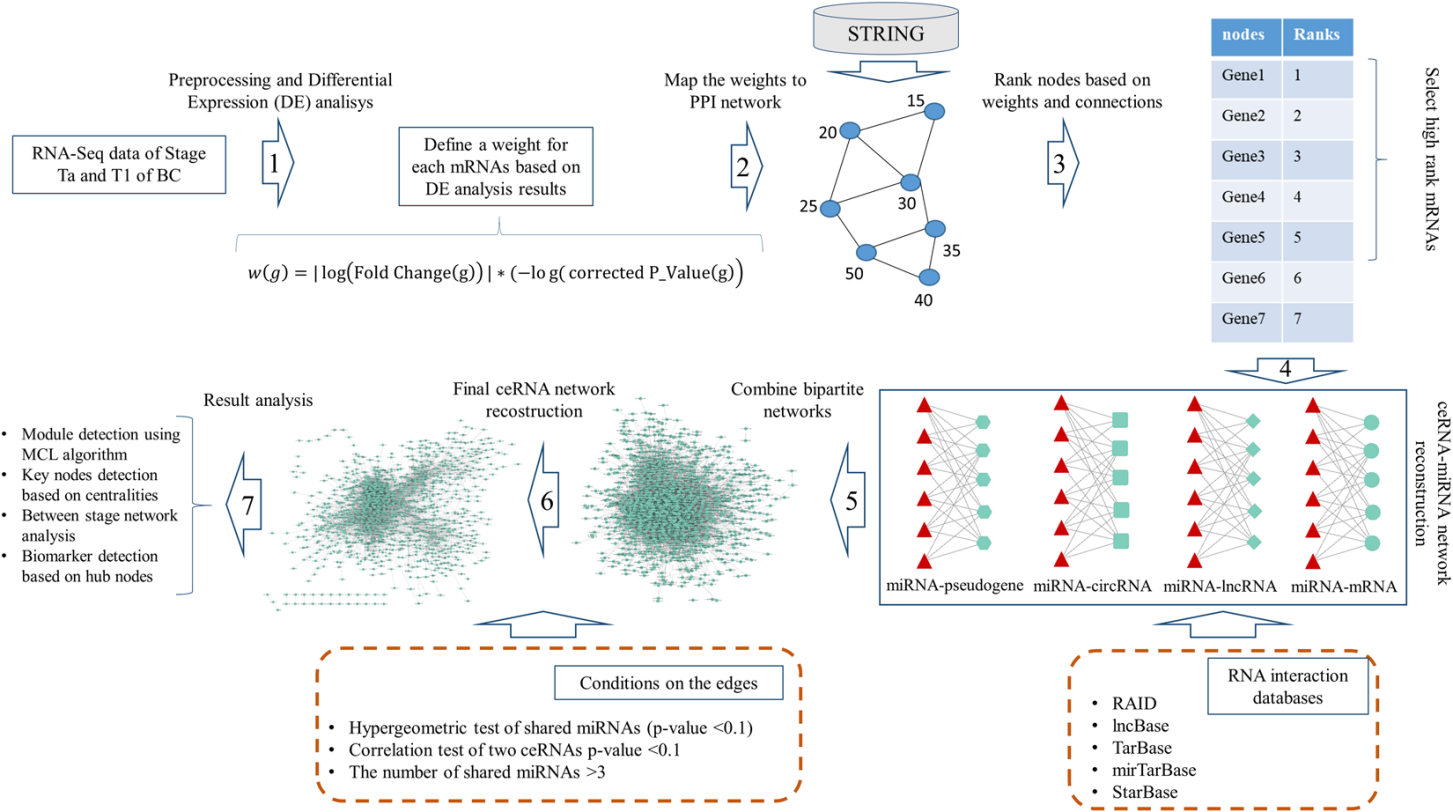
The overall process of our study.

The miRNAs that target the key mRNAs were extracted from the TarBase^73^ mirTarBase^74^ and RAID^75^ databases. In addition to the mRNA-miRNA bipartite network obtained from the key mRNAs and abovementioned databases, three other ceRNA-miRNA bipartite networks (lncRNA-miRNA, pseudogenes-miRNA, and circRNA-miRNA) were reconstructed based on the extracted miRNAs and the experimentally validated data from the RAID, lncBase^76^ and StarBase^77^ databases. The ceRNA-miRNA bipartite networks were merged and reconstructed to form a basic ceRNA-ceRNA interaction network. In this network, the nodes represent ceRNAs (mRNA, lncRNA, circRNA or pseudogene) and two ceRNAs are connected if they have common miRNAs in bipartite networks. All edges with less than three shared miRNAs were removed from the network. The hypergeometric test was applied to all ceRNA-ceRNA interactions based on Eq. 3^12^:

3 $$p \mbox{-} value=1-\sum _{i=0}^{s-1}\frac{(\begin{array}{c}{C}_{1}\\ i\end{array})(\begin{array}{c}T-{C}_{1}\\ {C}_{2}-i\end{array})}{(\begin{array}{c}T\\ {c}_{2}\end{array})}$$

where, *s* is the number of miRNAs shared between ceRNA1 and ceRNA2, *c*_1_ is the number of miRNAs targeting ceRNA1, *c*_2_ is the number of miRNAs targeting ceRNA2 and *T* is the total number of miRNAs in the human genome. All edges with a hypergeometric test p-value higher than 0.01 were removed from the basic network. Next, the normalized ceRNA count table was used to implement the Pearson correlation test for all interactions in the basic ceRNA-ceRNA interaction network. All edges with a correlation test p-value larger than 0.01 were removed from the network. Since the Pearson correlation test for each interaction was applied based on Ta and T1 samples separately, two final ceRNA-ceRNA interaction networks (Ta ceRNA network and T1 ceRNA network) were obtained for further analysis. Figure 9 shows the overall process of our study.

Additionally, we reconstructed between stage ceRNA network from Ta and T1 networks using the difference of edge sets. Cytoscape version 3.2^78^ was applied to visualize and reconstruct the network differences.

The Markov CLuster (MCL) algorithm^79^ via the ClusterMaker Cityscape application^80^ was used to detect modules in the networks.

### Enrichment analysis

The Gene Ontology (GO), disease and pathway enrichment analyses were performed using the ToppFun web tool^81^.

### Centrality calculation

The critical genes in the networks were predicted based on degree and betweenness centrality measures. The CytoNCA^82^ tool was applied to calculate centralities.

### Potential biomarker detection

For potential biomarker detection, 5, 10, 15 and 20 hub genes (genes with the highest degree) in the networks were selected. After that, all samples were clustered based on the expression pattern of each chosen gene set (hierarchical clustering with ward.D2 method^83^ and 1-Pearson correlation as the distance). The gene sets that significantly separated the Ta and T1 samples based on expression pattern were selected as candidate biomarker sets.

To validate biomarkers, we used SVM classifier and 5-fold cross-validation. Consequently, by calculating the Receiver Operating Characteristic (ROC) curve and Area Under the Curve (AUC) the best gene sets were selected as final potential biomarkers to detect BC in the early stage. The e1071^84^, plotROC^85^ and ggplot2^86^ R packages were used to apply the SVM algorithm and calculate and visualize ROC curves.

## Supplementary information

Supplementary file 1 (Supplementary Figures and Tables)

Supplementary file 2 (mRNAs Disease enrichment analysis results)

Supplementary file 3 (mRNAs BP enrichment analysis results)

Supplementary file 4 (mRNAs Pathway enrichment analysis results)

Supplementary file 5 (mRNAs PubMed enrichment analysis results)

Supplementary file 6 (DNR-Ta network modules enrichment analysis results)

Supplementary file 7 (DNR-T1 network modules enrichment analysis results)

Supplementary file 8 (PPR-Ta network modules enrichment analysis results)

Supplementary file 9 (PPR-T1 network modules enrichment analysis results)

Supplementary file 10 (MEG3 module enrichment analysis results)
