# Supplementary material for: Detection of novel biomarkers for early detection of Non-Muscle-Invasive Bladder Cancer using Competing Endogenous RNA network analysis
Source: Sci Rep. 2019 Jun 10;9:8434. doi: 10.1038/s41598-019-44944-3 (PMC6557814; doi:10.1038/s41598-019-44944-3)
Supplement: Supplementary file 1 — (Supplementary Figures and Tables) [file 41598_2019_44944_MOESM1_ESM.docx]

# **Detection of novel biomarkers for early detection of Non-Muscle-Invasive Bladder Cancer using Competing Endogenous RNA network analysis**

**Morteza Kouhsar^1^, Sadegh Azimzadeh Jamalkandi^2^, Ali Moeini^3^, Ali Masoudi-Nejad^1*^**

1. Laboratory of Systems Biology and Bioinformatics (LBB), Institute of Biochemistry and Biophysics, University of Tehran, Tehran, Iran
2. Chemical Injury Research Center, Systems Biology and Poisonings Institute, University of Medical Sciences Tehran, Tehran, Iran
3. Faculty of Engineering Sciences, College of Engineering, University of Tehran, Tehran, Iran

# Supplementary Figures and Tables


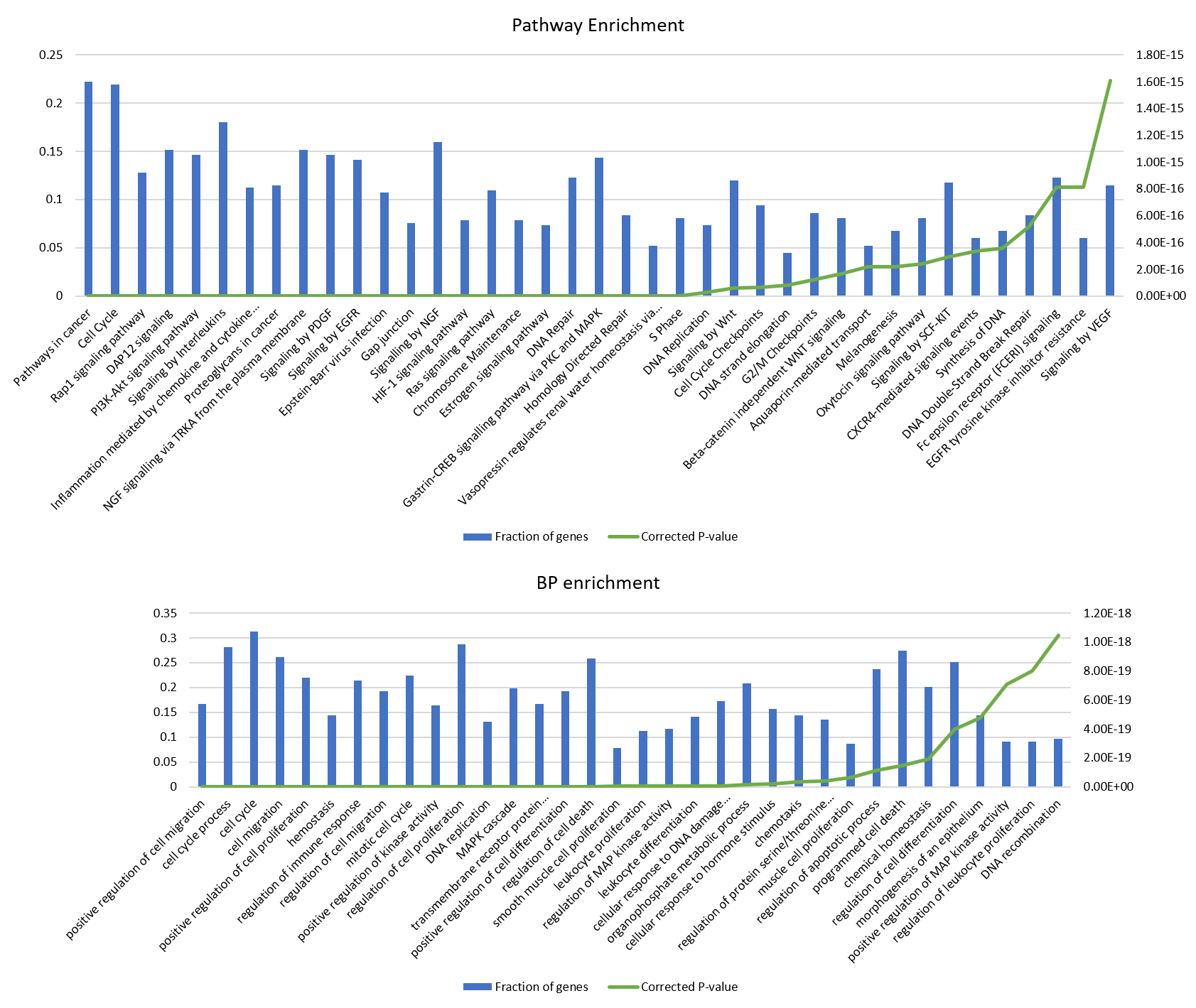


**Figure S1.** Some of the most critical enriched pathways and processes belonging to the mRNAs selected by PPR approach that they are not in the DEGs set.


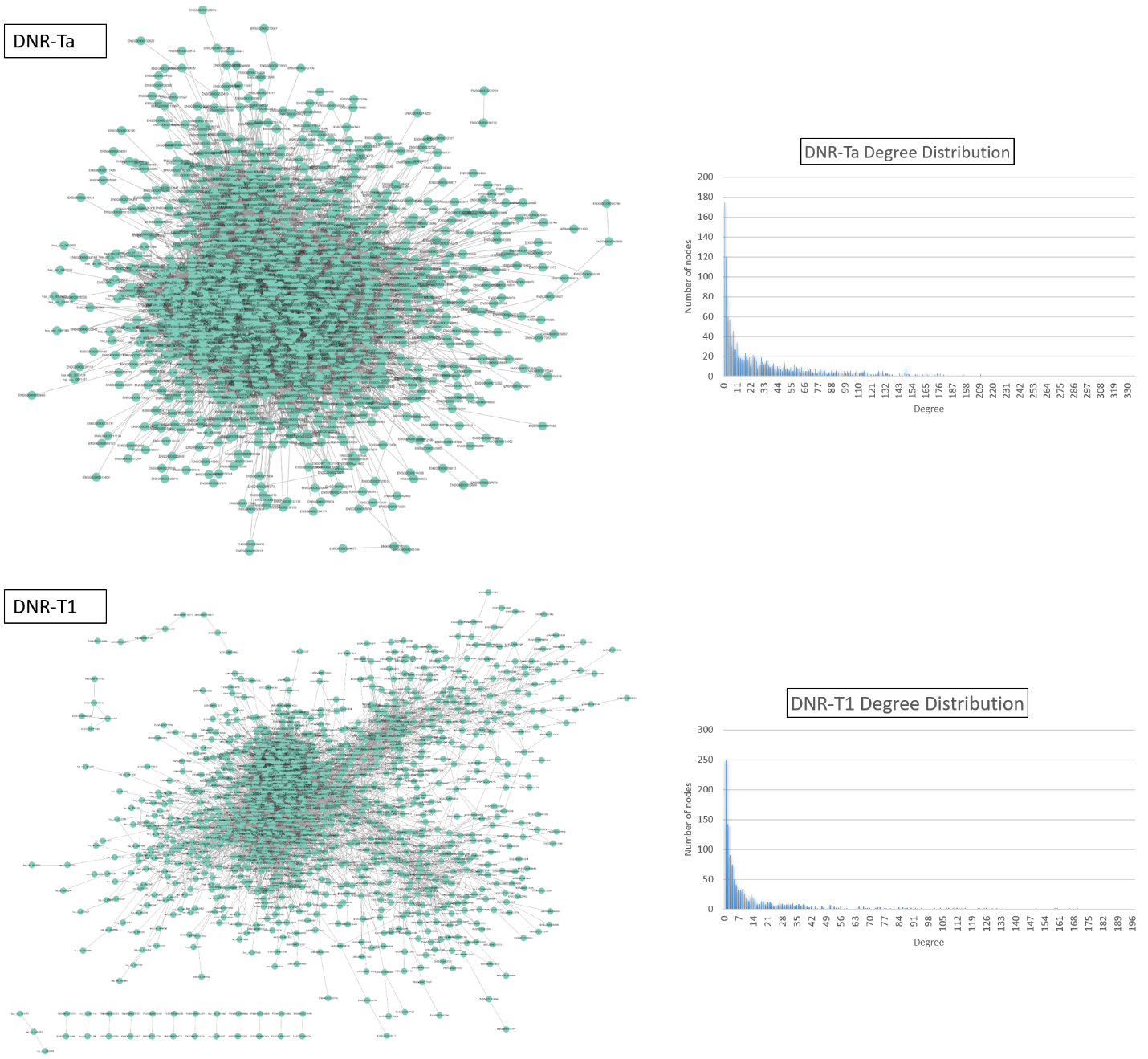


**Figure S2.** The ceRNA networks reconstructed based on the DNR approach.


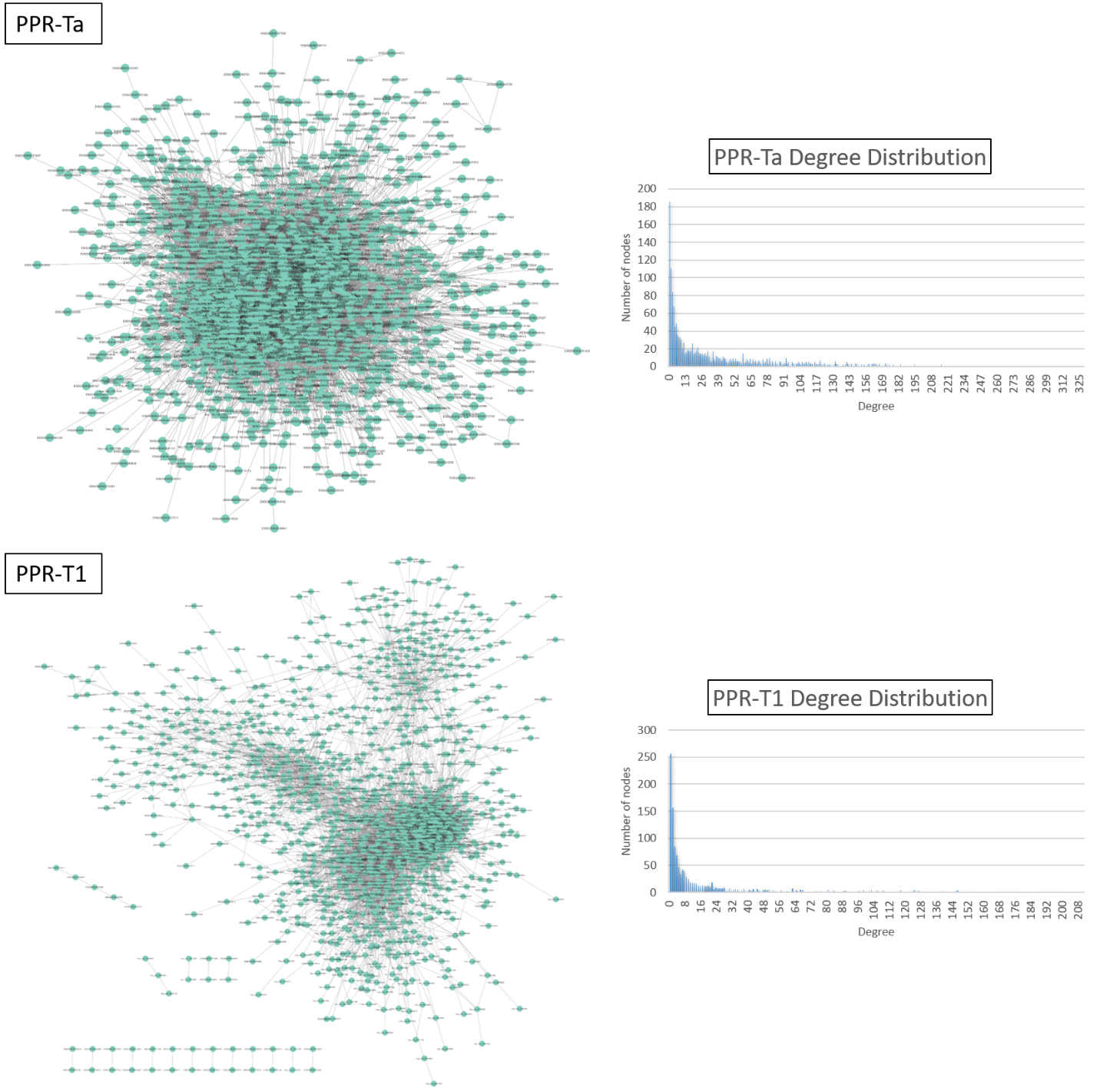


**Figure S3.** The ceRNA networks reconstructed based on the PPR approach.


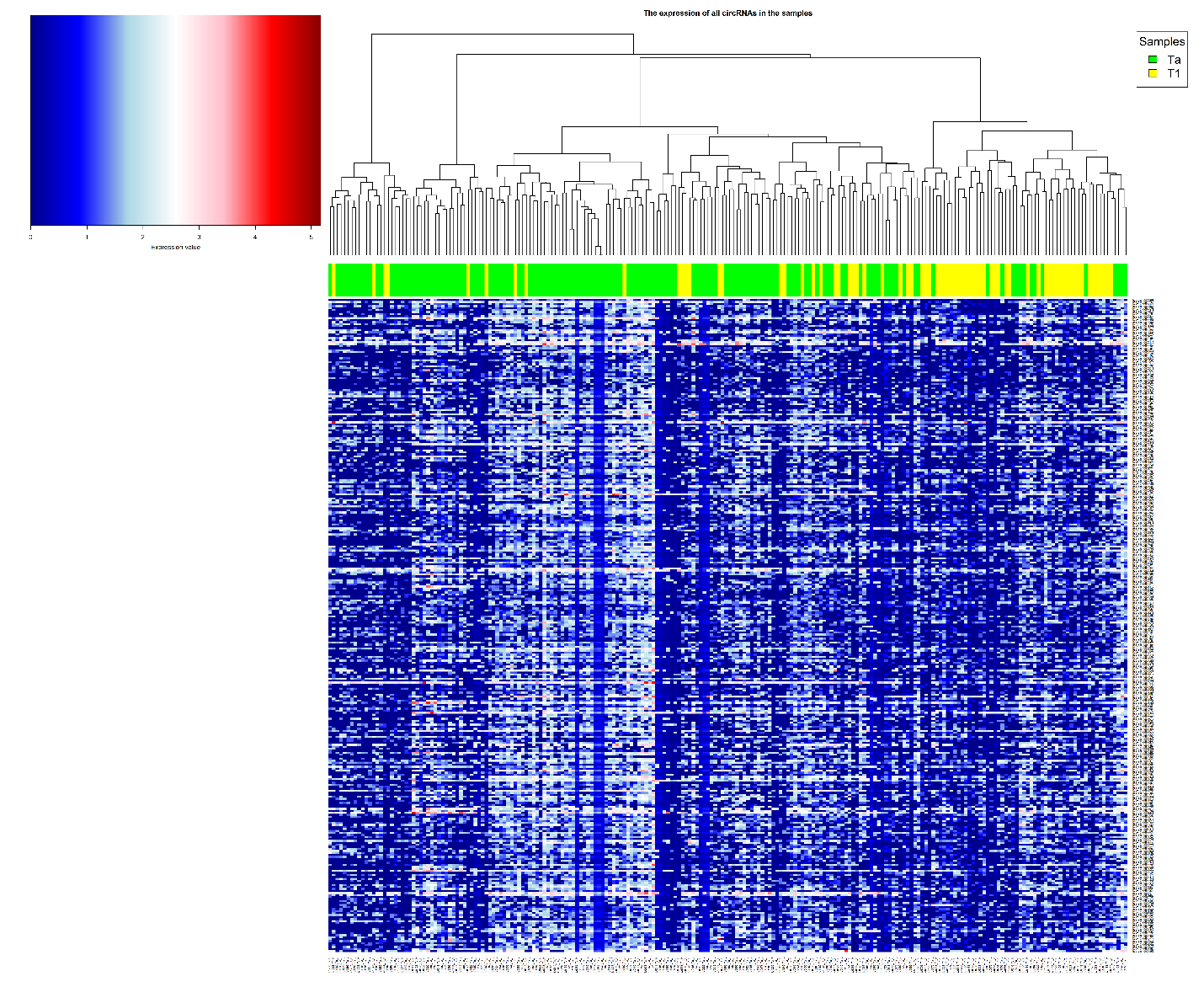


**Figure S4.** The expression pattern of all circRNAs in the ceRNA networks.


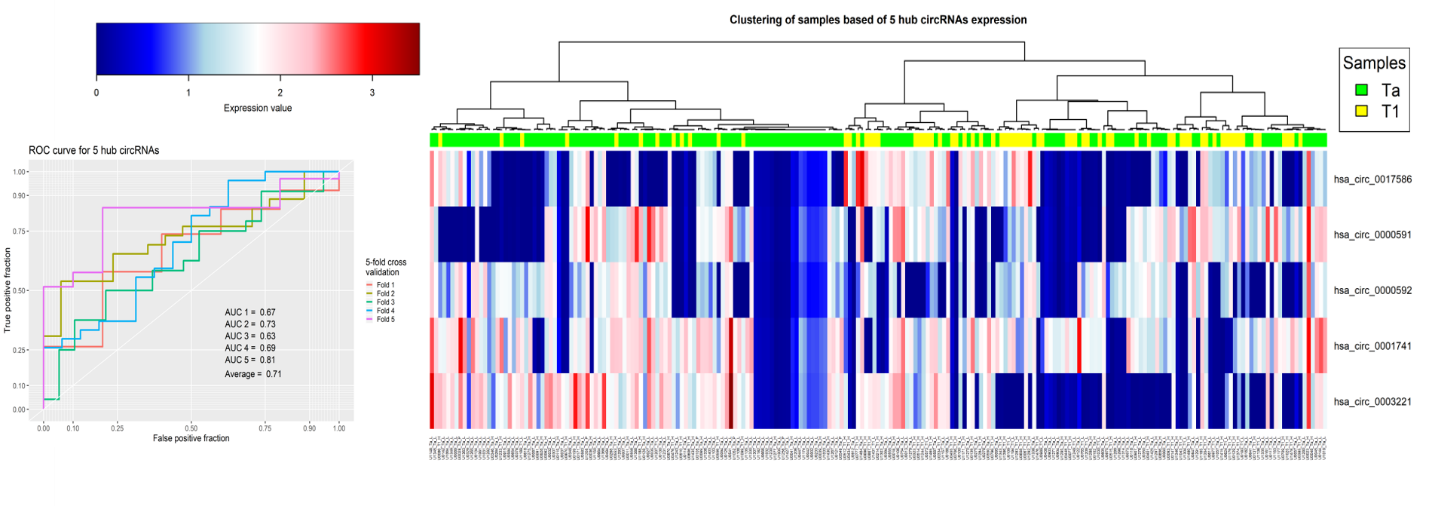


**Figure S5.** The clustering and validation results for five hub circRNAs as candidate biomarker in the networks.


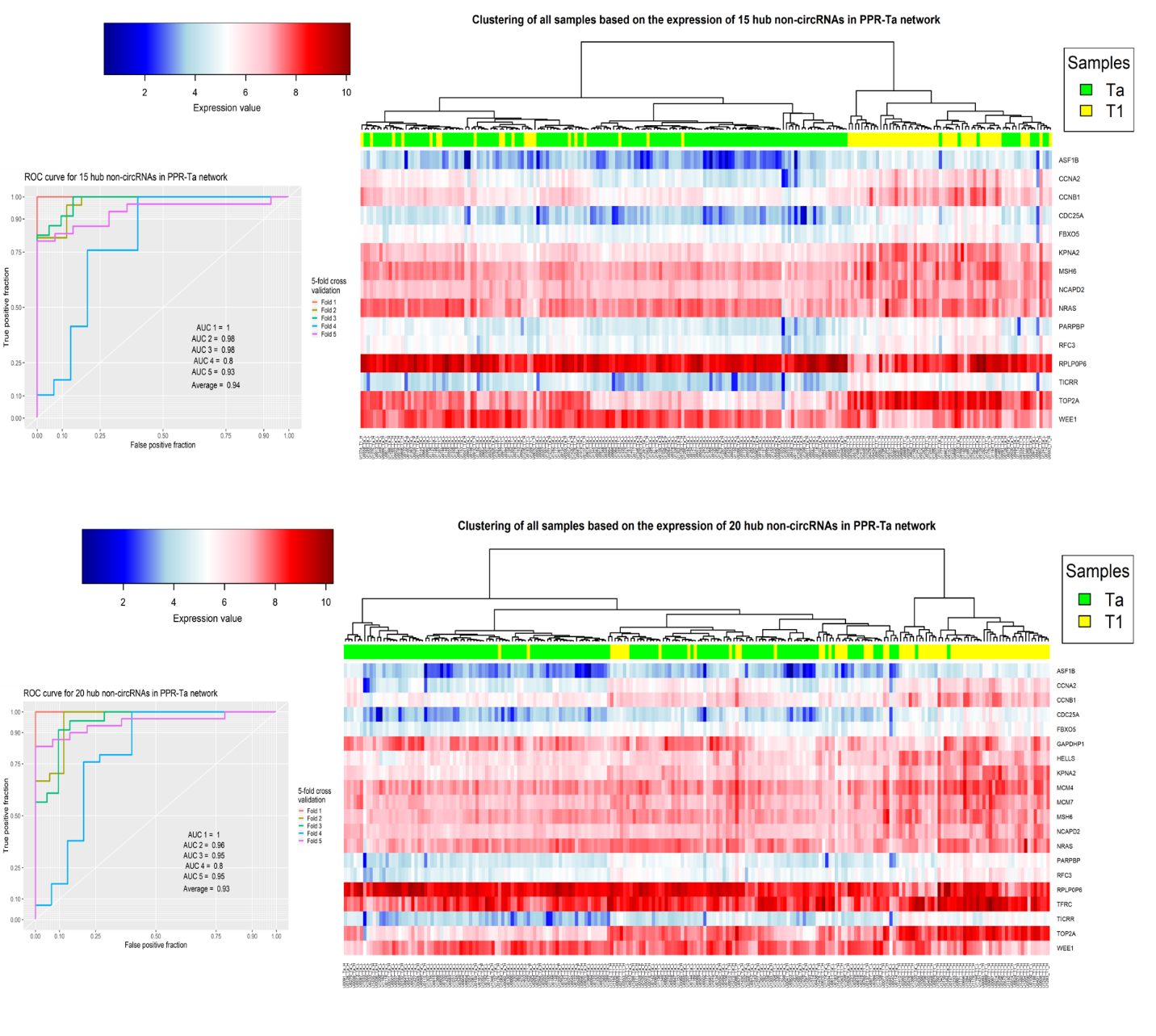


**Figure S6.** The clustering and validation results for 15 and 20 genes with the highest degree in DNR-Ta network.

**Table S1.** The cancer related genes found in MEG3 module.

| Gene name | Gene Type | network | Cancer type | Reported function in cancer | PMID |
| --- | --- | --- | --- | --- | --- |
| ADAMTS9-AS2 | lncRNA | DNR-Ta, DNR-T1,  PPR-Ta,  PPR-T1 | **Bladder** | Differentially expressed | 30170380  28389669 |
| BGN | coding |  | **Bladder** | Upregulated | 28459201 |
| CARMN | lncRNA | DNR-T1, PPR-T1, DNR-Ta | **Bladder** | suppresses bladder cancer development through inactivating Wnt/β-catenin pathway by modulating miR-1275/AXIN2 axis | 30471109 |
| CD34 |  | DNR-T1, PPR-T1 | **Bladder** | CD34 Microvessel density is associated with recurrence after BCG immunotherapy. |  |
| CMAHP | Pseudogene |  | leukemia | - | 23735562 |
| CXCL12 | coding | PPR-T1 | **Bladder** | CXCL12 mRNA expressions gradually increased in bladder cancer tissues | 26782415 |
| DAB2 | coding | DNR-Ta | **Bladder** | Decreased DOC-2/DAB2 expression seems to occur early in bladder tumorigenesis |  |
| DCN | coding | DNR-Ta,  PPR-T1,  PPR-Ta | **Bladder** | Downregulated, Tumor suppressor | 28459201 |
| DLG2 | coding | DNR-Ta | osteosarcoma | Tumor suppressor | 30093633 |
| FENDRR | lncRNA | DNR-Ta, DNR-T1,  PPR-Ta,  PPR-T1 | Renal cell carcinoma, non-small cell lung cancer (NSCLC), **Bladder** | Downregulation of FENDRR associates with poor prognosis of renal cell carcinoma, inhibits NSCLC cell growth and aggressiveness by sponging miR-761, Downregulated | 30655744  30556873  28389669 |
| GAS6-AS2 | lncRNA | DNR-Ta, PPR-Ta | **Bladder** | promotes bladder cancer proliferation and metastasis via GAS6-AS2/miR-298/CDK9 axis, up-regulated in bladder cancer tissues and positively correlated with tumour stages and poor prognosis. | 30394665 |
| HGF | coding | DNR-Ta, DNR-T1,  PPR-Ta,  PPR-T1 | **Bladder** | hepatocyte growth factor (HGF)-MET signaling is correlated with invasiveness of BCa cells | 28013036 |
| HOTAIR | lncRNA | PPR-Ta | **Bladder** | RNA-binding protein HuR promotes bladder cancer progression by competitively binding to the long noncoding HOTAIR with miR-1 | 28553126 |
| ITGB3 | Coding | DNR-T1, PPR-T1 | **Bladder** | MiR-320a down-regulation mediates bladder carcinoma invasion by targeting ITGB3 | 24443232 |
| KIT | coding | DNR-T1, PPR-T1 | **Bladder** | SCF/c-Kit signaling promotes the invasiveness of bladder cancer T24 cells | 24752098 |
| MEF2C-AS1 | lncRNA | DNR-Ta, PPR-Ta | Diffuse gastric cancer | knock-down of MEF2C-AS1 or FENDRR promoted aggressive tumor behaviors in in-vitro assays | 30005210 |
| MEG3 | lncRNA | DNR-Ta, DNR-T1,  PPR-Ta,  PPR-T1 | **Bladder** | suppresses the development of bladder urothelial carcinoma, inhibits cell migration and invasion | 29940769  30461333 |
| MIR99AHG | lncRNA | DNR-Ta, PPR-Ta | leukemia | Act as regulators of hematopoiesis and oncogenes in the development of myeloid leukemia | 25027842 |
| MITF | coding | DNR-T1, PPR-T1 | **Bladder** | Involved in c-Met/Akt/GSK-3β/Snail signaling and inhibition of the epithelial-mesenchymal transition in bladder cancer | 26844702 |
| NALCN-AS1 | lncRNA | DNR-Ta, PPR-Ta | Renal cell carcinoma | associated with overall survival | 30278113 |
| NR2F1-AS1 | lncRNA | DNR-Ta, PPR-Ta | Hepatocellular carcinoma | regulates hepatocellular carcinoma oxaliplatin resistance by targeting ABCC1 via miR-363 | 29602203 |
| PCAT19 | lncRNA | DNR-Ta, PPR-Ta, PPR-T1 | Prostate, **Bladder** | activate a subset of cell-cycle genes associated with Prostate cancer progression | 30033362  28389669 |
| PDGFB | coding | DNR-T1 | **Bladder** | - | 30397177 |
| PDGFRB | Coding | PPR-T1 | Non-muscle-invasive **bladder** cancer (NMIBC) | significantly correlated with the risk of 3-year recurrence of NMIBC, could serve as a non-invasive biomarker for predicting NMIBC recurrence | 24801713 |
| PTCH1 | Coding | DNR-Ta | **Bladder** | Upregulated | 21861243 |
| RAMP2-AS1 | lncRNA | DNR-Ta, PPR-TA | Glioblastoma | overexpression of RAMP2-AS1 reduced glioblastoma cell proliferation in vitro | 27784795 |
| RASSF8-AS1 | lncRNA | PPR-Ta | Non–Small-Cell Lung Carcinoma | Downregulated | 25590602 |
| SLC16A1-AS1 | lncRNA | PPR-Ta | **Bladder** | Involved in Bladder cancer invasiveness mediation | PMID is not available* |
| TEK | Coding | DNR-Ta | **Bladder** | - | 22607948 |
| TEX41 | lncRNA | DNR-Ta | Cervical | - | 30018982 |
| TNS1 | Coding | DNR-Ta, DNR-T1 | Non-muscle-invasive **bladder** cancer | Low levels of TNS1 have been associated with worsening-free survival in non-muscle invasive bladder cancer | 29150671 |
| TUBB6 | Coding | DNR-Ta,  PPR-Ta | Gastric | Upregulated | 26330360 |
| ZBTB16 | Coding | DNR-Ta | Prostate | Tumor suppressor | 29050363 |

* DOI:10.1158/1538-7445.AM2017-1895
